# *notch3* is essential for oligodendrocyte development and vascular integrity in zebrafish

**DOI:** 10.1242/dmm.012005

**Published:** 2013-05-29

**Authors:** Andreas Zaucker, Sara Mercurio, Nitzan Sternheim, William S. Talbot, Florence L. Marlow

**Affiliations:** 1 Department of Developmental and Molecular Biology, Albert Einstein College of Medicine, 1300 Morris Park Avenue, Bronx, NY 10461, USA; 2 Department of Developmental Biology, Stanford University School of Medicine, Beckman Center B300, 279 Campus Drive, Stanford, CA 94305, USA

## Abstract

Mutations in the human *NOTCH3* gene cause CADASIL syndrome (cerebral autosomal dominant arteriopathy with subcortical infarcts and leukoencephalopathy). CADASIL is an inherited small vessel disease characterized by diverse clinical manifestations including vasculopathy, neurodegeneration and dementia. Here we report two mutations in the zebrafish *notch3* gene, one identified in a previous screen for mutations with reduced expression of *myelin basic protein* (*mbp*) and another caused by a retroviral insertion. Reduced *mbp* expression in *notch3* mutant embryos is associated with fewer oligodendrocyte precursor cells (OPCs). Despite an early neurogenic phenotype, *mbp* expression recovered at later developmental stages and some *notch3* homozygous mutants survived to adulthood. These mutants, as well as adult zebrafish carrying both mutant alleles together, displayed a striking stress-associated accumulation of blood in the head and fins. Histological analysis of mutant vessels revealed vasculopathy, including: an enlargement (dilation) of vessels in the telencephalon and fin, disorganization of the normal stereotyped arrangement of vessels in the fin, and an apparent loss of arterial morphological structure. Expression of *hey1*, a well-known transcriptional target of Notch signaling, was greatly reduced in *notch3* mutant fins, suggesting that Notch3 acts via a canonical Notch signaling pathway to promote normal vessel structure. Ultrastructural analysis confirmed the presence of dilated vessels in *notch3* mutant fins and revealed that the vessel walls of presumed arteries showed signs of deterioration. Gaps in the arterial wall and the presence of blood cells outside of vessels in mutants indicated that compromised vessel structure led to hemorrhage. In *notch3* heterozygotes, we found elevated expression of both *notch3* itself and target genes, indicating that specific alterations in gene expression due to partial loss of Notch3 function might contribute to the abnormalities observed in heterozygous larvae and adults. Our analysis of zebrafish *notch3* mutants indicates that Notch3 regulates OPC development and *mbp* gene expression in larvae, and maintains vascular integrity in adults.

## INTRODUCTION

The Notch signaling pathway is widely conserved among metazoans. Vertebrate genomes contain several Notch paralogs, which control key developmental decisions at numerous stages and in diverse tissues and organs (Andersson et al., 2011; Gazave et al., 2009). The Notch pathway is ideally suited for signaling between adjacent cells because both the Notch receptor and its ligands are transmembrane proteins (Kopan and Ilagan, 2009). This feature allows Notch signals to assign different fates to adjacent cells with similar developmental potential. According to prevailing models, feedback mechanisms amplify small differences in the expression levels of Notch receptor and ligand in neighboring cells, resulting in active Notch signaling in one cell and suppression of Notch signaling in the other. This lateral inhibition mechanism directs binary fate decisions in many species and developmental contexts, including neurogenesis in *Drosophila* (Ruohola et al., 1991), development of the inner ear and the intestine in vertebrates (Cotanche and Kaiser, 2010; Crosnier et al., 2005; Haddon et al., 1998a; Heath, 2010), and development of the vertebrate vasculature (Gridley, 2010). In the developing vasculature, Notch signaling inhibits endothelial cells from adopting a tip cell fate during sprouting angiogenesis (Sainson et al., 2005). Accordingly, inhibition of Notch signaling in zebrafish and mouse leads to increased sprouting and branching of vessels due to supernumerary tip cells (Leslie et al., 2007; Siekmann and Lawson, 2007). The Notch pathway also confers arterial identity to endothelial cells and vascular mural cells [e.g. vascular smooth muscle cells (VSMCs) and pericytes] (Domenga et al., 2004; Lawson et al., 2001).

In *Drosophila*, *notch* pathway mutants have neurogenic phenotypes characterized by an excess of neuronal cells (Campos-Ortega, 1985). The role of Notch signaling in limiting neurogenesis is conserved in vertebrates (Lewis, 1996). Mutants disrupting vertebrate pathway components also display neurogenic phenotypes (Imayoshi et al., 2010; Yoon et al., 2008). These phenotypes have been attributed to excessive differentiation of neural progenitor cells (NPCs) into neurons, which depletes the progenitor pool in *notch* pathway mutants. Thus, Notch regulates the balance between neuronal differentiation and maintenance of progenitor cell fates, including of radial glia. Notch also drives differentiation of other glial cell types (e.g. Müller glia of the retina), and Notch activity is thought to broadly control the balance between neurogenesis and gliogenesis (Pierfelice et al., 2011). In addition, Notch activity promotes expansion of oligodendrocyte precursor cells (OPCs), while inhibiting their terminal differentiation (Park and Appel, 2003; Wang et al., 1998). Outside of the nervous system, the Notch pathway contributes to the maintenance of other progenitor and stem cell populations, such as Leydig progenitor cells of the testis and melanocyte stem cells (Moriyama et al., 2006; Tang et al., 2008).

**TRANSLATIONAL IMPACT**

**Clinical issue**

Mutations in the human *NOTCH3* gene cause cerebral autosomal dominant arteriopathy with subcortical infarcts and leukoencephalopathy (CADASIL). CADASIL is an inherited disease characterized by progressive degeneration of small blood vessels, which leads to a diverse set of clinical manifestations including vasculopathy, neurodegeneration and dementia. None of the existing CADASIL mouse models fully recapitulates the human disease. In light of the variability among patients and the limitations of currently available *NOTCH3* and CADASIL animal models, the mechanism by which *NOTCH3* mutations cause CADASIL symptoms – including vascular, white matter and other disease phenotypes – remain to be clarified.

**Results**

In this study, zebrafish *notch3* mutants were analyzed to gain insight into the mechanisms underlying CADASIL pathophysiology. The authors report that mutations in *notch3* reduce the expression of *myelin basic protein* (*mbp*) and the number of oligodendrocyte precursor cells (OPCs). Despite displaying an early neurogenic phenotype, some *notch3* homozygous mutants survived to adulthood. These mutants, as well as adult zebrafish carrying both mutant alleles together, displayed a striking stress-associated accumulation of blood in the head and fins. The authors’ histological analysis revealed vasculopathy in the mutants, including enlarged vessels in the brain and peripheral circulatory system and an apparent loss of arterial morphological structure. Moreover, ultrastructural analysis revealed signs of vessel deterioration in *notch3* mutants, including gaps in the arterial wall and the presence of blood cells outside of vessels. Subsequent examination of Notch pathway target genes indicated that Notch3 acts via a canonical Notch signaling pathway to promote normal vessel structure. In *notch3* heterozygotes, expression of *notch3* and its target genes was elevated, indicating that alterations in gene expression occur in response to partial loss of Notch3.

**Implications and future directions**

These analyses of zebrafish *notch3* mutants indicate that Notch3 regulates OPC development and *mbp* gene expression in larvae, and plays a conserved role in maintaining vascular tone and vessel integrity in adults. Changes in gene expression and cell death in the CNS were observed specifically in *notch3* heterozygotes, suggesting that a partial loss of Notch3 function could contribute to aspects of the pathophysiology of CADASIL. These changes were not detected using previously reported animal models that completely lack *Notch3* expression. Zebrafish *notch3* mutants provide a valuable tool for future screens aiming to identify Notch signaling modifiers that affect the phenotype, and for studies of the activity of CADASIL mutant alleles and the signaling and cellular mechanisms that contribute to Notch3-associated pathologies.

Mutations in the human *NOTCH3* gene cause CADASIL syndrome (cerebral autosomal dominant arteriopathy with subcortical infarcts and leukoencephalopathy). CADASIL is a small vessel disease that has been diagnosed in more than 500 families worldwide (Hervé and Chabriat, 2010). Clinical symptoms comprise migraine with aura, leukoaraiosis, subcortical infarcts, mood disturbances, apathy and dementia (Hervé and Chabriat, 2010). Many pathological mutations in CADASIL result in an odd number of cysteines in one of the EGF repeats of NOTCH3, which is thought to cause aggregation of the NOTCH3 extracellular domain (NECD) in individuals with CADASIL (Joutel et al., 1997; Opherk et al., 2009). The majority of individuals with CADASIL display leukoaraiosis and ischemic events that are particularly prominent in the white matter of the CNS, a region characterized by low blood flow that consists primarily of glial cells and myelinated axons (Lee et al., 2012). In the CNS, oligodendrocytes form myelin, a multi-lamellar sheath that allows for rapid impulse propagation by saltatory conduction in the vertebrate nervous system. Damage to oligodendrocytes and demyelination of axons can cause or contribute to neurodegenerative pathology, as occurs in the CNS in multiple sclerosis (MS) (Prineas and Parratt, 2012). The demyelination of axons might contribute to the cerebral atrophy seen in individuals with CADASIL (Viswanathan et al., 2006); however, axonal damage and atrophy in the white matter has also been viewed as a secondary defect to vascular disruption.

Histopathological analysis revealed that CADASIL is a vasculopathy of small cerebral and systemic arteries (Baudrimont et al., 1993; Ruchoux et al., 1995). In CADASIL, the VSMCs show signs of degeneration and eventually are lost. Endothelial dysfunction and ultrastructural endothelial changes also have been reported in CADASIL. Although vascular changes are observed all along the arterial tree, and in liver, muscle, spleen and skin, organ failure is only apparent in the brain and retina (Robinson et al., 2001; Ruchoux et al., 1995). It is not clear why widespread arteriopathy does not cause more global organ defects.

In an effort to better understand CADASIL pathologies, *Notch3* knockout, knock-in and transgenic mouse models have been developed (Arboleda-Velasquez et al., 2011; Arboleda-Velasquez et al., 2005; Joutel, 2011; Joutel et al., 2004; Krebs et al., 2003; Monet-Leprêtre et al., 2009; Ruchoux et al., 2003). The defective constrictive responses upon experimental challenge by proximal middle cerebral artery occlusion and the enlarged arteries with thinner walls that are observed in *Notch3* knockout mice indicate roles for Notch3 in arterial VSMC specification and maturation in tail vessels, and in maintaining vascular tone (Arboleda-Velasquez et al., 2008; Belin de Chantemèle et al., 2008; Domenga et al., 2004; Krebs et al., 2003). Although arteriopathies in the tail and, when challenged, cortical vessels (Arboleda-Velasquez et al., 2008) have been observed in knockout mice, important pathologies and hallmarks of CADASIL – including Notch3 ectodomain deposits, leukoencephalopathy and lacunar infarcts – have not been found. Nevertheless, *Notch3* knockout mice show a higher susceptibility for ischemic stroke (Arboleda-Velasquez et al., 2008). None of the existing CADASIL mice fully recapitulates the human disease. In light of this variability among the available *Notch3* and CADASIL animal models, the mechanism by which *Notch3* mutations cause CADASIL symptoms is not known, and the mechanism and extent of interdependence of the vascular, white matter and other disease phenotypes remain to be clarified.

In a genetic screen to identify genes that are required for the development of myelinated axons in zebrafish, *st51* was discovered as a mutation that reduced *myelin basic protein* (*mbp*) mRNA expression in the CNS (Pogoda et al., 2006). Here we show that *st51* disrupts the zebrafish *notch3* gene. Analysis of *notch3^st51^* and an insertional allele of *notch3* revealed that *notch3* is required for OPC development and myelin gene expression during larval development. At later stages, *mbp* expression increased in the CNS of *notch3* mutants, and some homozygous mutants survived to adulthood. In these adult mutants, we observed bleeding in response to mild stress. Histological and electron microscopy (EM) analyses revealed enlarged vessels in the telencephalon and fins, which indicated that blood accumulation was due to dilation and leaking of the vessels in *notch*3 mutant adults. Our studies of zebrafish *notch3* mutants provide evidence that Notch3 plays a conserved role in maintaining vascular tone and vessel integrity in vertebrate adult vessels. The presence of oligodendrocyte abnormalities before vascular pathology provides evidence that *notch3* might function in parallel in myelination and vasculogenesis.

## RESULTS

### Identification of mutations in *notch3*

**Fig. 1.**
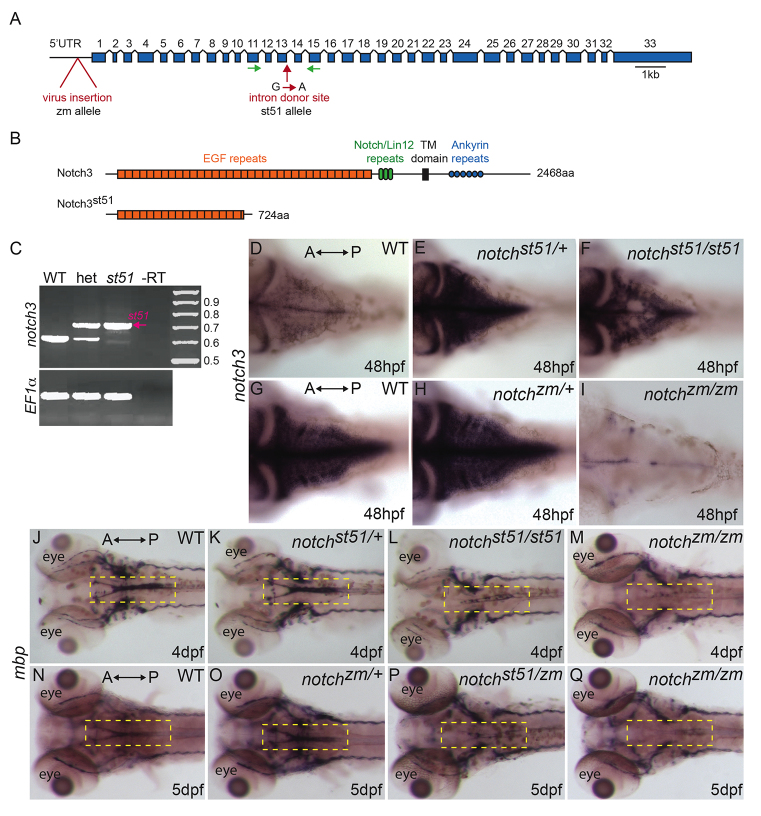
**Mutations in *notch3* reduce *mbp* mRNA expression.** (A) Schematic depicting the *notch3* gene and the locations of the retroviral insertion (*zm* allele) and of the *st51* mutation. Introns are not to scale. (B) Schematic of the wild-type Notch3 protein domains and the truncated protein produced owing to the *st51* mutation. (C) RT-PCR of the wild-type (WT) and *st51 notch3* transcripts. (D–I) In (D,G) wild-type zebrafish, (E) *notch3^st51/+^* and (H) *notch3^zm/+^* heterozygotes, and (F) *notch3^st51/st51^* mutants, *notch3* transcripts were detected in the CNS. In contrast to heterozygotes and *notch3^st51/st51^* mutants, *notch3* expression was barely detectable in the CNS of (I) *notch3^zm/zm^* mutants. Panels D–F and G–I are age-matched siblings from *st51* and *zm* clutches, respectively. (D–F) Short staining reaction. (G–I) Long staining reaction. (J–Q) Expression of *mbp* was similar in (J,N) wild-type zebrafish and (K,O) *notch3^st51/+^* or *notch3^zm/+^* heterozygotes, but was reduced in the CNS (compare yellow dashed areas in the hindbrain) of (L) *notch3^st51/st51^* and (M,Q) *notch3^zm/zm^* homozygotes and (P) *notch3^st51/zm^* transheterozygotes, indicating that the *st51* and *zm* mutations failed to complement. (D–Q) Dorsal views. Anterior (A) and posterior (P) are indicated.

In a screen for zebrafish mutants with abnormal myelination, Pogoda et al. identified *st51* as a mutation that reduced *mbp* expression in the CNS at 5 dpf (Pogoda et al., 2006). Previous mapping experiments localized the *st51* mutation to linkage group 3 (Pogoda et al., 2006). To identify the gene disrupted by *st51*, we conducted additional mapping experiments and localized the mutation to an interval bordered by the simple sequence length polymorphism (SSLP) markers Z3725 and Z20058, which contains the *notch3* gene (Woods et al., 2005). Sequence analysis of genomic DNA from *st51* mutants revealed a G-to-A transition in the mutant allele that disrupts the splice donor site after exon 13 in the *notch3* gene (Fig. 1A). We detected no recombination between this lesion and the *st51* mutation among 348 meioses (data not shown), indicating that *st51* was tightly linked to *notch3*. Reverse transcriptase PCR (RT-PCR) analysis of RNA isolated from wild-type and mutant larvae at 3 dpf revealed that the *notch3* pre-mRNA was aberrantly spliced in *st51* homozygotes, such that intron 13was retained in the majority of mutant transcripts (Fig. 1B,C). Retention of this intron in the predominant mutant transcript caused truncation of the predicted *notch3* open reading frame in the extracellular region, after the 17th EGF repeat (Fig. 1B). In addition, a minority of the mutant *notch3* mRNAs contained aberrantly spliced sequences that result in partial deletions of exon 13. Sequence analysis identified two such deletions, a 15 bp deletion that maintained the reading frame, and a 103 bp deletion that induced a frameshift, truncating the predicted *notch3* reading frame after the 16th EGF repeat (Fig. 1B,C).

To further investigate the function of *notch3*, we obtained and examined a commercially available *notch3* allele, *ZM_197263*, produced by insertional mutagenesis. The *ZM_197263* mutation (referred to as ‘*zm*’ hereafter) contains a retroviral insertion in the 5′UTR of the *notch3* gene, 158 nucleotides upstream of the open reading frame (Fig. 1A). In wild-type animals, *notch3* was expressed throughout the nervous system, including in the hindbrain (Fig. 1D). Compared with wild type (Fig. 1D,G; *n*=5 each), *notch3^st51/+^* and *notch3^zm/+^* heterozygous siblings (Fig. 1E,H; *n*=11 each) and *notch3^st51/st51^* mutants (Fig. 1F; *n*=7) showed enhanced expression of *notch3* in the CNS, a finding we return to below. In contrast, little if any *notch3* mRNA was detectable in *notch3^zm/zm^* mutants (Fig. 1I; *n*=9), indicating that *notch3^zm^* is a strong loss-of-function mutation.

To examine the phenotype of *notch3^zm/zm^* mutants and determine whether *st51* and *zm* fail to complement, we examined *mbp* expression in early larvae (Fig. 1J–Q). Expression of *mbp* was similar to wild type in *notch3^st51/+^* heterozygotes (Fig. 1K), but was reduced in the CNS of *notch3^st51/st51^* homozygotes (Fig. 1L) and *notch3^zm/zm^* homozygotes (Fig. 1M). The reduction of *mbp* expression in the CNS of *notch3^zm/zm^* mutants, *notch3^st51/st51^* homozygotes and *notch3^st51/zm^* transheterozygotes at 5 dpf (Fig. 1N–Q and data not shown) indicated that the *st51* and *zm* mutations failed to complement. Moreover, the reduction seemed to be more severe in *notch3^zm/zm^* mutants, suggesting that the *notch3^st51^* mutation might have more residual *notch3* function than does *notch3^zm^*. On the basis of mapping data, the identification of lesions that disrupt the splicing or expression level of *notch3* and complementation analysis, we conclude that both the *st51* and *zm* mutations disrupt the function of *notch3*. Phenotypic and molecular studies suggest that *notch3^zm^* is a null or near-null mutation, and that *notch3^st51^* is a hypomorphic allele that probably retains weak *notch3* function. The residual Notch3 activity in *notch3^st51/st51^* homozygotes is probably provided by the minor splice variant with the 15 bp deletion that maintains the reading frame in these mutants. These results demonstrate that *notch3* is essential for normal expression of *mbp* in the larval CNS.

### Transient reduction in OPCs in *notch3* mutant larvae

**Fig. 2.**
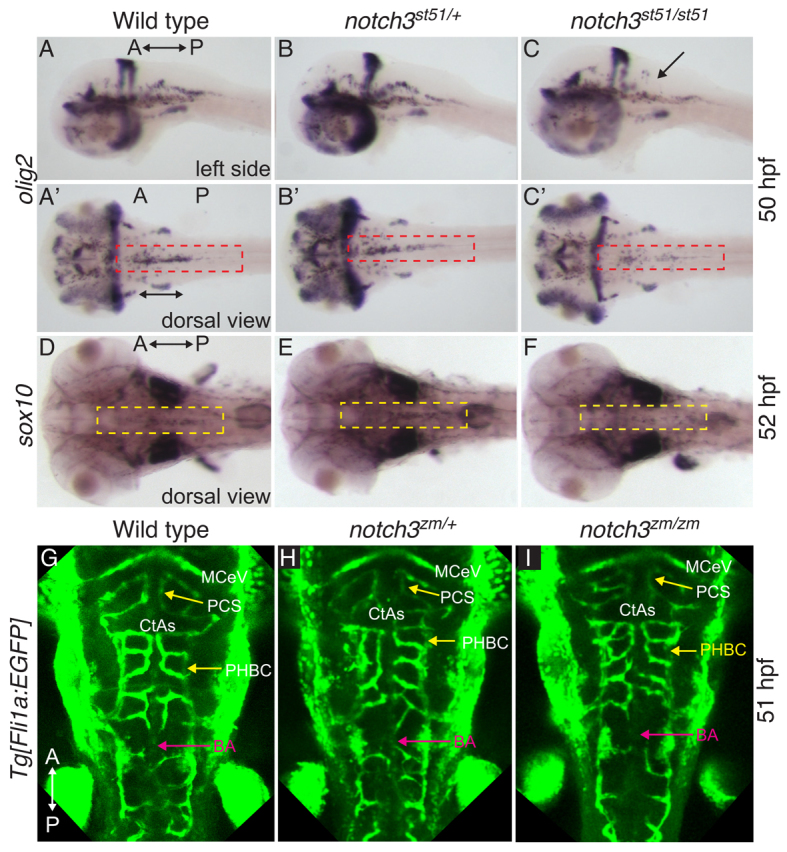
**Transient reduction in OPCs in *notch3* mutants at stages at which vessel patterning is intact.** (A–C) *olig2* at 50 hpf was similar in (A,A′) wild type and (B,B′) *notch3^st51/+^* heterozygotes, but was reduced in OPCs (compare red dashed areas) of (C,C′) *notch3^st51/st51^* mutants. Arrow indicates region devoid of *olig2*-expressing cells. (D–F) *sox10* expression at 52 hpf was comparable in (D) wild type and (E) *notch3^st51/+^* heterozygotes, but was reduced in the midline of the hindbrain (compare yellow dashed areas) in (F) *notch3^st51/st51^* mutants. (A–C) Lateral views. (A′–C′,D–I) Dorsal views. (G–I) Vessel pattern at the anatomical level is intact in *notch3^zm/zm^* mutants. Max projection images of *Tg[fli1a:EGFP]* labeled vasculature of (G) wild type (*n*=9), (H) *notch3^zm/+^* heterozygotes (*n*=9) and (I) *notch3^zm/zm^* mutants (*n*=5). CtAs, central arteries; PHBC, primordial hindbrain channel; BA, basilar artery; MCeV, mid-cerebral vein; PCS, posterior connecting segment. The orientation of the anterior (A) and posterior (P) axis is indicated.

Mutational analysis shows that *notch3* is essential for normal expression of *mbp* mRNA in the CNS of zebrafish larvae (Pogoda et al., 2006) (and this work). To investigate the possibility that an early deficit in oligodendrocytes underlies reduced *mbp* expression in *notch3* mutants, we examined *oligodendrocyte transcription factor 2* (*olig2*)*,* which is expressed in OPCs and motor neurons (Park et al., 2002; Park et al., 2007; Shin et al., 2003; Takebayashi et al., 2000; Zannino and Appel, 2009). At 24 hpf, expression of *olig2* was comparable between wild type, *notch3^st51/+^* heterozygotes and *notch3^st51/st51^* homozygotes (data not shown). However, *olig2* expression was reduced in OPCs of *notch3^st51/st51^* mutants at 50 hpf (Fig. 2C,C′; *n*=5) compared with wild type (Fig. 2A,A′; *n*=2) and *notch3^st51/+^* heterozygotes (Fig. 2B,B′; *n*=8). At 52 hpf, expression of *sox10*, an independent marker of cells of the oligodendrocyte lineage, was also reduced in the midline of the hindbrain in *notch3^st51/st51^* mutants (Fig. 2F, compared with 2D,E). These results suggested that a partial loss of OPCs resulted in the reduced expression of *mbp* in the CNS of *notch3* mutants. This analysis indicates that *notch3* is required for OPC development and myelin gene expression in the embryo and early larva. Despite these defects at earlier stages, some *notch3* mutants achieve normal levels of *mbp* expression in the hindbrain by 7 dpf (data not shown).

In individuals with CADASIL, loss of myelin accompanies and has been proposed to be caused by vascular defects (Okeda et al., 2002), and adult *notch3* knockout mice have vascular defects. Although swim bladder inflation was delayed at 5 days post-fertilization (dpf), no overt evidence of edema or compromised circulation was detected morphologically at 3–5 dpf (stages when OPC defects were apparent among progeny) in *notch3^st51/st51^* or *notch3^zm/zm^* mutants from five independent heterozygous incrosses. To more closely examine vessel patterning, we conducted microangiography and crossed the *notch3* mutations into the *Tg[fli1a:EGFP]* background (Lawson and Weinstein, 2002b). As compared with wild-type genotypes, microangiography (supplementary material Fig. S1A–C) and expression of *Tg[fli1a:EGFP]* (Fig. 2G–I and supplementary material Fig. S2) indicated that, at the anatomical level, overall sprouting, vessel branching and morphogenesis were intact in *notch3* mutants at stages before and after OPC deficits were already apparent. Consistent with *Tg[fli1a:EGFP]* analysis and microangiography, vascular markers [*deltaC* (*dlC*), *ephrinB2* (*efn2ba/ephB2*), *fms-related tyrosine kinase 4* (*flt4*) and *disabled homolog 2* (*dab2*)] were indistinguishable between wild type, *notch3^zm/+^* heterozygotes and *notch3^zm/zm^* mutants at 28 hpf (supplementary material Fig. S3A–L), providing additional evidence that, at the anatomical level, arterial venous identity and arterial venous patterning of the vasculature is intact at stages when OPCs and oligodendrocytes are disrupted in *notch3* mutants.

### Neurogenic phenotype of *notch3* mutants and apoptosis in *notch3* heterozygotes

**Fig. 3.**
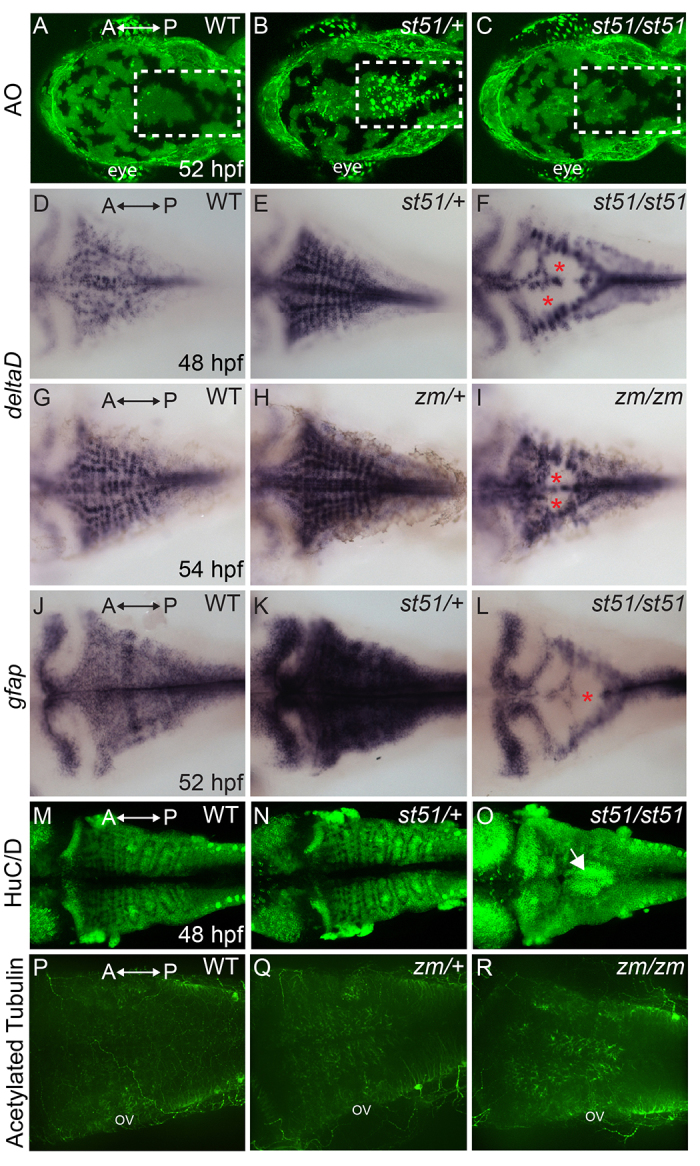
**Apoptosis in *notch3* heterozygotes and neurogenic phenotype of *notch3* mutants.** (A–C) Acridine orange (AO)-labeled embryos. The hindbrains (white dashed areas) of (A) wild type (WT) and (C) *notch^st51/st51^* mutants were largely devoid of AO-positive cells at 52 hpf. In contrast, AO-positive cells were abundant in the hindbrain region of (B) *notch3* heterozygotes. (D–I) Dorsal views of *deltaD* expression. (D,G) In wild type, *deltaD* mRNA was abundant in the CNS. *deltaD* expression was more pronounced in (E) *notch3^st51/+^* and (H) *notch3^zm/+^* heterozygotes. *deltaD* expression was robust in lateral regions and showed gaps in medial regions (asterisks) of (F) *notch3^st51/st51^* and (I) *notch3^zm/zm^* mutants. (J,K) Expression of another progenitor marker, *gfap*, which marks neural progenitor cells, was more robust in (K) *notch3^st51/+^* heterozygotes compared with in (J) wild type. (L) In *notch3^st51/st51^* mutants, *gfap* was present in lateral regions and showed medial gaps (asterisk). (M–O) HuC/D, a marker of post-mitotic neurons, expression was comparable in (M) wild type and (N) *notch3^st51/+^* heterozygotes, but was more robust in the medial hindbrain (arrow) of *notch3^st51/st51^* mutants. (P-R) Acetylated-tubulin-labeled (P) wild-type (*n*=5), (Q) *notch3^zm/+^* heterozygotes (*n*=11) and (R) *notch3^zm/zm^* mutant (*n*=9) larvae. (P-R) Max projections of confocal images. ov, otic vesicle. All panels are dorsal views, anterior (A) is to the left and posterior (P) is to the right.

To investigate whether the reduction of OPCs in *notch3* mutants was due to loss of progenitors via cell death, we used Acridine Orange (AO) to label cells undergoing apoptosis. At 52 hpf, AO staining was similar in the CNS of wild-type siblings (Fig. 3A; *n*=12) and *notch3^st51/st51^* mutants (Fig. 3C; *n*=8), indicating that the reduction of OPCs in *notch3* mutants was not due to cell death. Because apoptosis did not account for reduced OPCs in mutants, we examined the expression of progenitor and differentiation markers. During neuronal development, *deltaD* expression in precursors prevents neighboring *notch*-expressing cells from adopting neuronal fates (Haddon et al., 1998b; Hans and Campos-Ortega, 2002; Mahler et al., 2010; Takke et al., 1999). We observed abundant *deltaD* mRNA (Fig. 3D–I) in lateral regions of *notch3^st51/st51^* (Fig. 3F; *n*=11) and *notch3^zm/zm^* (Fig. 3I; *n*=2 at 48 hpf and 4 at 54 hpf) mutants, with holes in *deltaD* expression in medial regions similar to the gaps in *notch3* and *notch1a* expression that were present in *notch3^st51/st51^* mutants (Fig. 1F; *n*=11 and supplementary material Fig. S4). To further investigate the development of neural progenitors, we examined *gfap* expression at 52 hpf, which marks radial glial progenitors at this stage (Fig. 3J–L). As we observed for *notch3* and *deltaD*, there were medial gaps in *gfap* expression in *notch3* mutants (Fig. 3L; *st51: n*=8; *zm: n*=9), indicating that OPCs might be reduced owing to aberrant differentiation of medial progenitors.

To investigate the possibility that the reduction of OPCs results from a neurogenic phenotype in *notch3* mutants, we examined HuC/D, which marks post-mitotic neurons. In contrast to wild type (Fig. 3M) and *notch3* heterozygotes (Fig. 3N and data not shown), *notch3* mutants had abundant HuC/D expression (Fig. 3O and data not shown) and acetylated tubulin staining (compare with Fig. 3P–R) in medial regions of R3/R5, consistent with the possibility that OPCs are reduced because progenitors are inappropriately allocated to neuronal fates in *notch3* mutants. In other regions of the embryo, major axonal projections were comparable between wild type and *notch3* mutants (supplementary material Fig. S5).

A striking feature of the foregoing analysis is that *notch3* heterozygotes expressed highly elevated levels of Notch pathway components, including *deltaD* [Fig. 3E (80% of *notch3^st51/+^* heterozygotes; *n*=26); Fig. 3H (*n*=9 *notch3^zm/+^* heterozygotes at 48 hpf and 6 *notch3^zm/+^* heterozygotes at 54 hpf)] and *notch3* itself (Fig. 1E), and the progenitor marker *gfap* (Fig. 3K). In addition, AO staining revealed elevated cell death in the CNS of *notch3^st51/+^* (Fig. 3B; *n*=12) and *notch3^zm/+^* (*n*=2) heterozygotes that was not seen in wild type or homozygous mutants (Fig. 3A, *n*=12; Fig. 3C, *n*=8). Despite these changes in gene expression and cell death, *notch3* heterozygotes were fully viable and fertile. Indeed, even some *notch3* homozygous mutants achieved normal levels of *mbp* expression in the hindbrain by 7 dpf (data not shown) and survived to adulthood.

### Compromised vascular tone in *notch3* mutant adults

**Fig. 4.**
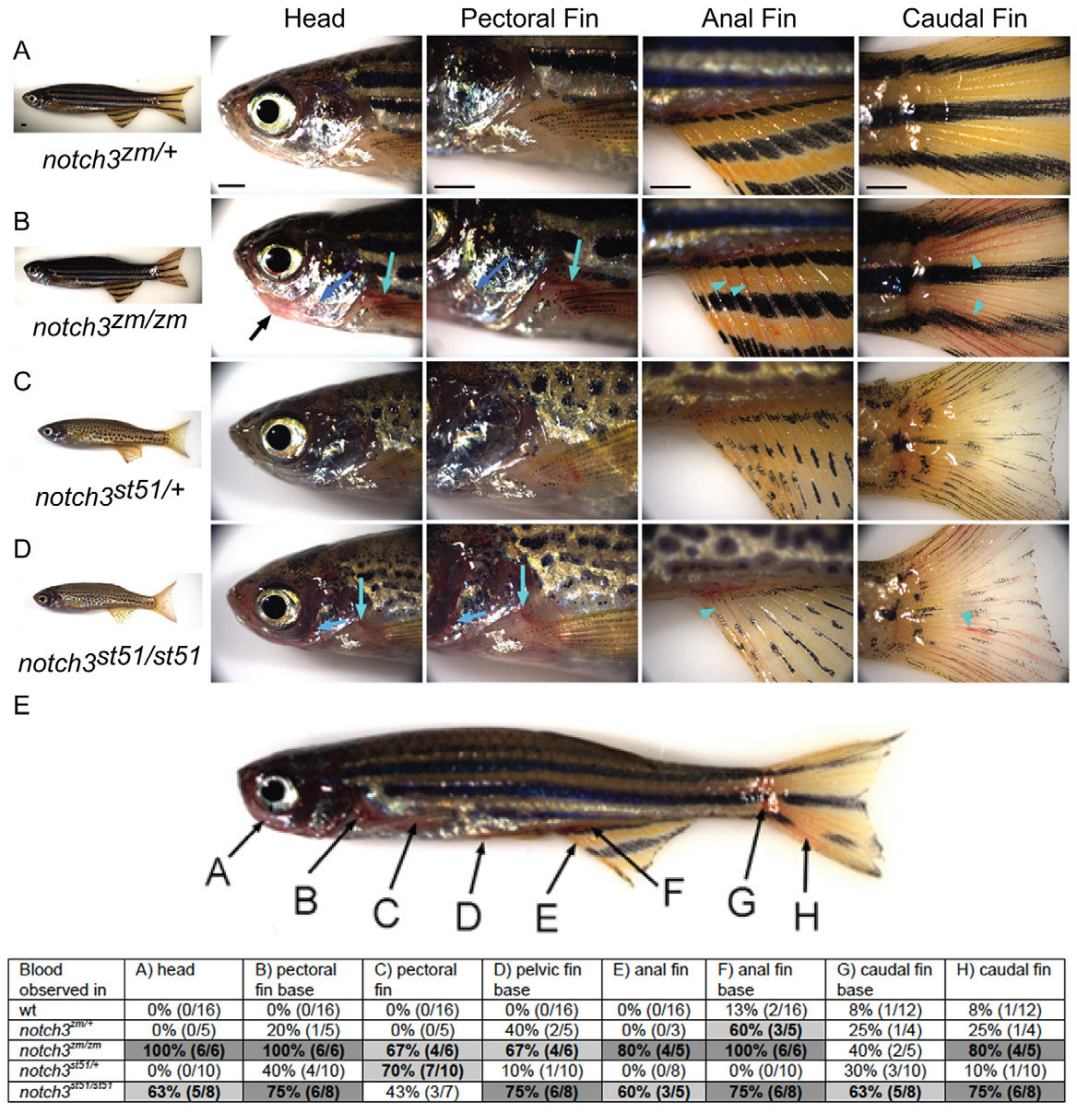
**Vascular phenotype of *notch3* mutants.** (A–D) Lateral views of (A) *notch3^zm/+^* heterozygotes, (B) *notch3^zm/zm^* mutants, (C) *notch3^st51/+^* heterozygotes and (D) *notch3^st51/st51^* mutants. (B) *notch3^zm/zm^* mutant adults show craniofacial defects (black arrow) and accumulation of blood (light and dark blue arrows and arrowheads) in the head, pectoral anal and caudal fins. (E) Quantification of the bleeding phenotypes. Degree of shading indicates penetrance of phenotype: white cell in the table indicates less than 50% of animals showed the phenotype, light gray indicates that 50–75% of individuals were affected, and dark gray denotes greater than 75% of animals had the phenotype.

Despite the reduced numbers of OPCs in *notch3^st51/st51^* and *notch3^zm/zm^* mutants, a subset of the mutants recovered from these early zygotic phenotypes and survived to adulthood. Homozygous *notch3^st51/st51^* and *notch3^zm/zm^* males that survived to adulthood were fertile. However, in response to mild stress (such as moving the tank from the rack, or mating), the homozygous mutant adults displayed an accumulation of blood within the head and fins that was not observed in wild-type or heterozygous siblings (Fig. 4). In *notch3^zm/+^* (Fig. 4A,E) and *notch3^st51/+^* (Fig. 4C,E) heterozygous siblings, blood was evident at the base of the fins, but did not extend into the caudal fin rays. In contrast, blood extended from the base to the tip of the fins in *notch3^zm/zm^* (Fig. 4B,E) and *notch3^st51/st51^* (Fig. 4D,E) homozygotes and *notch3^st51/zm^* transheterozygotes (data not shown), indicating that vascular tone might be compromised in *notch3* mutant adults. As observed for the reduction in *mbp* expression in the larvae, *notch3^zm/zm^* mutants showed more pronounced phenotypes compared with *notch3^st51/st51^* mutants.

**Fig. 5.**
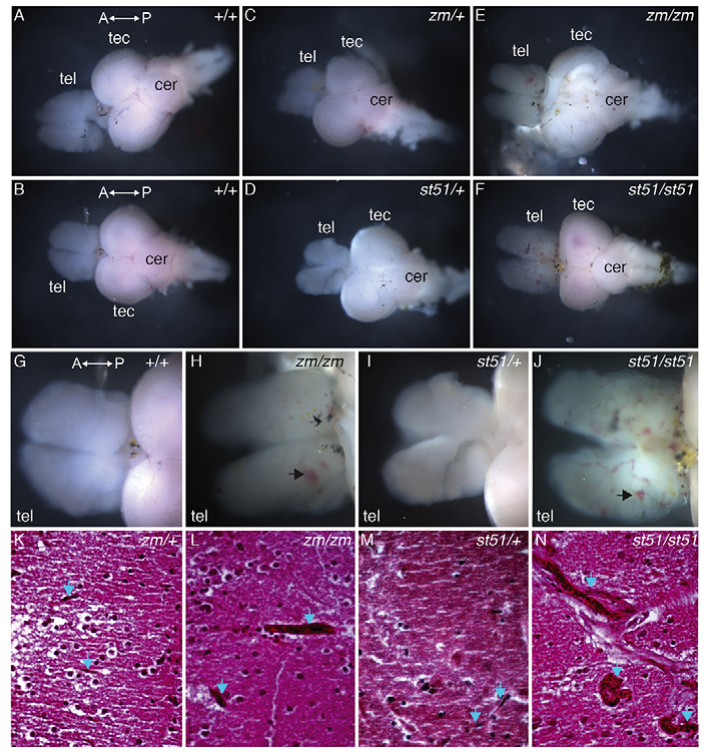
**Vessels are more prominent in the telencephalon of *notch3* mutants compared with wild type.** (A–F) Overview images of dissected adult brains. (G–J) High-magnification images of the telencephalon. Compared with (A,B,G) wild-type and (C,D,I) heterozygous genotypes, vessels are more prominent in the telencephalon of (E,H) *notch3^zm/zm^* and (F,J) *notch3^st51/st51^* mutants. (G–J) Large vessels and accumulation of blood (arrows) are apparent in mutant telencephalons. (K–N) H&E-stained sections of the medial telencephalon of (K) *notch3^zm/+^*, (L) *notch3^zm/zm^*, (M) *notch3^st51/+^* and (N) *notch3^st51/st51^* mutants. Small vessels traverse the tissue in heterozygotes, whereas large dilated vessels are present in homozygous mutant telencephalon. Blue arrows denote vessels. tel, telencephalon; tec, tectum; cer, cerebellum. The orientation of the anterior (A) and posterior (P) axis is indicated. (K–N) Scale bars: 20 μm.

CADASIL is a vasculopathy of small cerebral and systemic arteries caused by mutations in NOTCH3 (Baudrimont et al., 1993; Ruchoux et al., 1995). We conducted histopathological analysis to investigate whether adult zebrafish *notch3* mutants showed any evidence of vasculopathy in the brain. Compared with wild-type (Fig. 5A,B,G) and heterozygous (Fig. 5C,D,I) genotypes, vessels were more prominent in the telencephalon of *notch3^zm/zm^* (Fig. 5E,H) and *notch3^st51/st51^* mutants (Fig. 5F,J). In contrast, the tectum of wild-type zebrafish had highly branched large- and small-diameter vessels (Fig. 5A), whereas the tectum of *notch3* mutants was covered with small-diameter vessels and capillaries (Fig. 5E,F). Histological analysis of hematoxylin and eosin (H&E)-stained sections within the medial telencephalon confirmed the presence of small vessels (probably capillaries) traversing the tissue of *notch3^zm/+^* and *notch3^st51/+^* heterozygotes (Fig. 5K,M). In contrast, abnormally large vessels were present in *notch3^zm/zm^* and *notch3^st51/st51^* mutant telencephalon (Fig. 5L,N), indicative of vasculopathy in *notch3* mutant brain tissue.

### Defective organization and vasodilation of vessels in *notch3* mutants

**Fig. 6.**
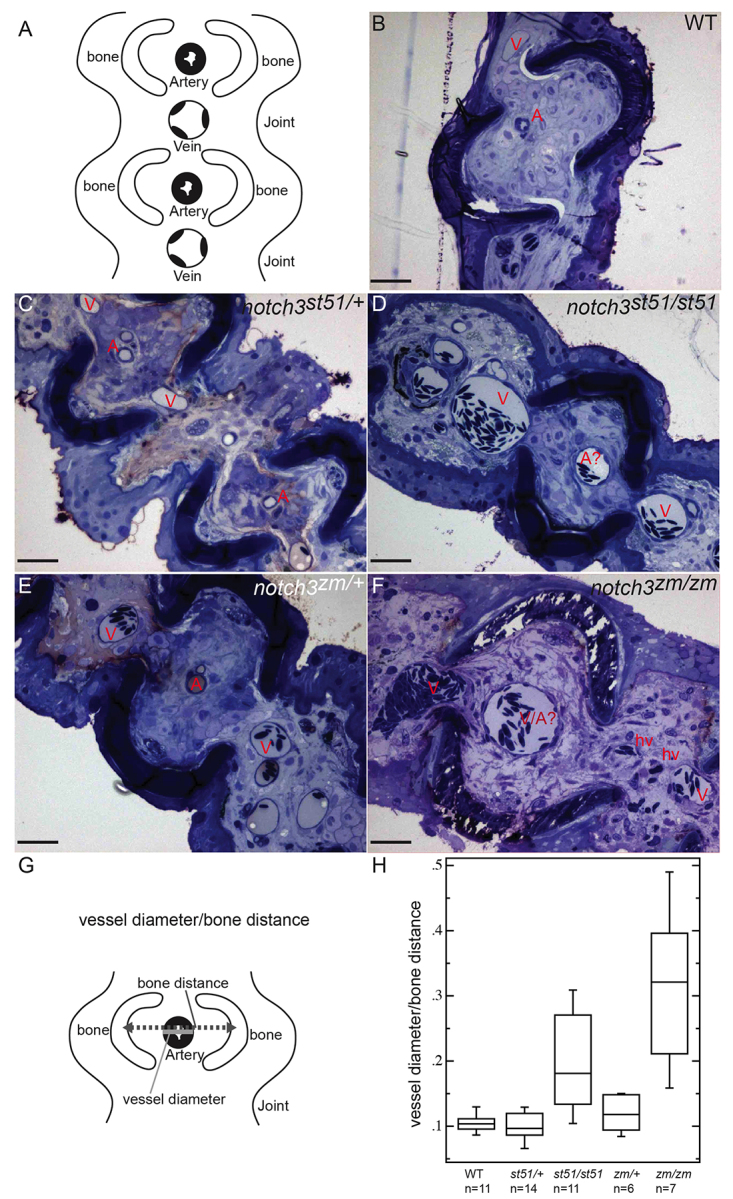
**Distension of vessels in *notch3* mutants.** (A) Schematic depicting cross-section and the ordered arrangement of arteries within the bony regions and larger caliber veins within the joints of the fin rays observed in (B) wild type, (C) *notch3^st51/+^* and (E) *notch3^zm/+^* heterozygotes. This pattern is moderately disrupted in (D) *notch3^st51/st51^* mutants and severely disrupted in (F) *notch3^zm/zm^* mutants. (B–F) Toluidine-blue-stained sections. Scale bars: 50 μm. A, artery; A?, probable artery based on position; V, vein; hv, hemorrhaged vessel. (G) Schematic depicting the strategy for quantification of vessel defects (detailed in the Materials and Methods). (H) Box plot of results reveals significantly larger vessels within the bony segments of the fin of *notch3* mutants compared with wild-type adults. ANOVA results: mean squares between=6.7821×10^−2^; mean squares error=3.4565×10^−3^; *P*<0.0001.

To further investigate the basis for the accumulation of blood in *notch3* mutants, we conducted histological analysis of caudal fin tissue isolated from *notch3* mutants. Wild-type fins had the expected highly ordered reiterative structure of dermal, vascular and nervous tissues, with veins occupying the joints between and arteries within bony regions (Fig. 6A,B; *n*=2). This ordered pattern was largely intact in *notch3^zm/+^* and *notch3^st51/+^* heterozygotes (Fig. 6C,E; *n*=2 each). By contrast, in *notch3^st51/st51^* mutants (Fig. 6D; *n*=2) veins were not limited to the joints and extended into the bony regions, which were co-occupied by multiple distended vessels. Similar but more severe phenotypes were observed in *notch3^zm/zm^* mutants (Fig. 6F; *n*=2). Even with higher magnification, it was difficult to unambiguously identify arteries in the *notch3^zm/zm^* mutant fins (Fig. 6F).

In order to quantitatively assess the vessel defects given the difficulty in distinguishing arteries from veins in *notch* mutants, we determined the ratio of the vessel diameter within the bony segments of the fin ray, where arteries are typically located, to the distance between the bones (Fig. 6G). This ratio was not significantly different between wild-type and *notch3^st51/+^* or *notch3^zm/+^* heterozygotes (Fig. 6H). In contrast, *notch^st51/st51^* and *notch3^zm/zm^* mutants showed statistically significant distension of the vessels residing within bony regions of the fin ray, such that vessels in *notch3^zm/zm^* mutants showed a threefold increase in relative diameter.

**Fig. 7.**
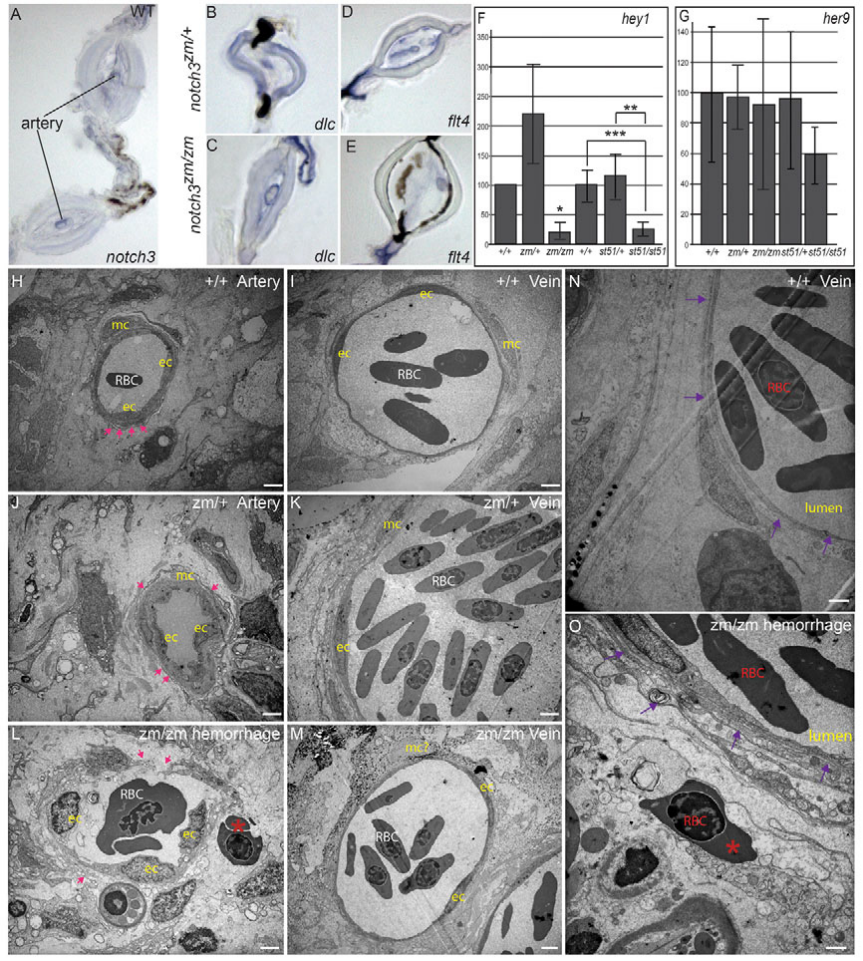
**Expression of *notch3,* Notch targets, and arterial and venous markers in adult fin, and analysis of compromised vessel integrity and hemorrhage of vessels in *notch3* mutants.** (A) Consistent with the vascular phenotypes of *notch3* mutants, we detected *notch3* expression in association within arteries of adult wild-type (WT) fin. (B) *deltaC* is expressed in the arteries within the bony segments, but not in the veins, which are located in the joints of *notch3^zm/+^* heterozygotes fins as expected. (C) In *notch3^zm/zm^* mutants, *deltaC* is also expressed in the enlarged vessels (note the enlarged lumen) within bony segments but not in the veins; thus, arterial and venous identity does not require *notch3*. (D,E) No difference in the pattern of *flt4* expression between (D) *notch3^zm/+^* heterozygotes and (E) *notch3^zm/zm^* mutant fins was detected, although the mutant vessels were distended. (F,G) qRT-PCR of Notch target expression in caudal fin. (F) qRT-PCR *hey1.* (G) qRT-PCR *her9. y*-axis indicates % of expression normalized to WT. (H–O) TEM analysis. (H) Wild-type artery. Pink arrows indicate dense plaques. (I) Wild-type vein. (J) Intact dense plaques surrounded arteries of *notch3^zm/+^* heterozygotes and (K) veins show normal morphology, but appear slightly enlarged. (L) Breaks in the walls and reduced dense plaques (pink arrows) of *notch3^zm/zm^* mutant vessels with features reminiscent of arteries. Blood cells (red asterisk) are located outside of the damaged vessel. (M) *notch3^zm/zm^* mutant veins are comparable to those of *notch3^zm/+^* heterozygotes. (N) Wild-type vein. The purple arrows in N and O indicate the vessel wall. (O) Hemorrhage and inflammation of *notch3^zm/zm^* vessel. Note the red blood cell (red asterisk in O) outside of the compromised vessel wall indicated by purple arrows. Scale bars: 1 μm. ec, endothelial cell; mc, vascular mural cells; RBC, red blood cell. *n=2; **P=0.06; ***P=0.02.

Consistent with the vascular phenotypes of *notch3* mutants, *notch3* expression was prominent in arteries of adult fin (Fig. 7A). In *notch3* mutants, the vessels within bony regions, where arteries normally reside, showed morphological features that were more reminiscent of veins. To investigate whether the *notch3* mutant vessels within bony segments showed aberrant morphology because they had lost their arterial identity, we examined the expression of *deltaC*, a marker of arterial identity. As in *notch3^zm/+^* heterozygotes (Fig. 7B), *notch3^zm/zm^* mutants (Fig. 7C) expressed *deltaC* in the enlarged vessels within bony segments. Importantly, *deltaC* was not expressed in the vessels that run adjacent to the bony segments, indicating that those vessels kept their venous identity. We also examined *flt4*, which was expressed in arteries and veins within the fin of *notch3^zm/+^* heterozygotes (Fig. 7D) and *notch3^zm/zm^* mutants (Fig. 7E). These analyses indicate that, although the mutant arteries displayed morphological features of veins, their arterial identity was intact in *notch3^zm/zm^* mutants.

Previous studies of mouse *Notch3* knockout mice suggest distinct mechanisms by which Notch3 functions. One proposed mechanism involves the regulation of canonical Notch targets such as *Hey* and *Hes* genes (Arboleda-Velasquez et al., 2008), whereas the second points to a newly identified pathway whereby Notch3 activates unknown targets (Domenga et al., 2004). Moreover, human CADASIL mutations show a differing ability to bind Jagged1 ligand and to activate canonical Notch targets (e.g. RBP-Jκ) (Joutel et al., 2004). To investigate the pathway underlying compromised vascular tone in zebrafish *notch3* mutants, we surveyed the expression of canonical Notch targets in mutant fins (Fig. 7F,G). We found that the canonical *notch* target *hey1* was significantly reduced in *notch3^zm/zm^* and *notch3^st51/st51^* mutant fins (Fig. 7F), and that levels of *her9* were not significantly changed (Fig. 7G). These results indicate that Notch3 acts via a mechanism that involves the activation of canonical Notch targets to promote vascular tone and integrity.

### Compromised vessel integrity leads to hemorrhage of vessels in *notch3* mutants

To investigate whether the accumulation of blood in the fins of *notch3* mutants was due to compromised vascular tone or reflected a more severe defect (hemorrhaging of the vessels), we used EM to examine the vascular ultrastructure. In wild type (Fig. 7H,I) and *notch3^zm/+^* heterozygotes (Fig. 7J,K), arteries and veins were readily distinguishable, albeit that veins seemed to be larger in *notch3^zm/+^* heterozygotes, suggesting a mild defect in vascular tone. In *notch3^zm/zm^* mutants the vessels were severely dilated and arteries were difficult to distinguish from veins (Fig. 7L,M and data not shown). Mural cells associated with vessels were easily identifiable in wild-type and heterozygous genotypes, but could not be unambiguously identified in *notch3* mutants. Moreover, gaps in the vessel walls and the presence of blood cells outside of the vessels indicated that the pooling of blood in the fins of *notch3^zm/zm^* mutants was probably due to both loss of vascular tone and hemorrhage of the *notch3* mutant vessels (compare Fig. 7N and 7O).

## DISCUSSION

The Notch pathway is highly conserved and is widely used to mediate cell fate decisions in embryonic and adult tissues (reviewed in Kopan and Ilagan, 2009). Mutations disrupting human *NOTCH3* cause CADASIL, an inherited small vessel disease associated with neurodegeneration and cognitive decline with advanced patient age (Hervé and Chabriat, 2010). Prevailing models indicate that cognitive decline might be secondary to vascular pathology, but this remains an open question because the existing mouse knockins of common patient *NOTCH3* alleles recapitulate some but not all of the CADASIL pathologies. The two reported *Notch3* knockout mouse models are susceptible to stroke when challenged, but no other neural deficits have been reported. Studies of these knockout mouse mutants point to opposing models of Notch3 function in establishing vascular structure. Based on their analysis of one knockout mutation, Domenga et al. presented evidence for a vascular defect and argue for a non-canonical mechanism (Domenga et al., 2004), whereas Arboleda-Velasquez et al. did not report vascular structure defects in a different knockout strain and argued that a canonical pathway was important for the regulation of gene expression in vascular smooth muscle cells (VSMCs) (Arboleda-Velasquez et al., 2011; Arboleda-Velasquez et al., 2008). Although the mouse analyses present different views of the role of *notch3* in establishing vascular structure, our analysis of zebrafish *notch3* mutants provides strong evidence that *notch3* has a conserved function in sustaining mature vessel structure and maintaining vascular integrity, probably via a canonical pathway, as indicated by reduced *hey1* in *notch3* mutants. In addition, our studies of zebrafish *notch3* mutants provide evidence that *notch3* regulates OPC development and *mbp* gene expression in larvae and promotes mural cell and artery maturation in adults. Our analysis of neural development reveal that *notch3* mutant larvae have a neurogenic phenotype and that *notch3* heterozygous larvae have abnormalities in development of the CNS that are not seen in homozygous mutants.

We demonstrate that *notch3* is essential for normal differentiation of neural progenitors during embryogenesis. In the developing hindbrain of *notch3* mutants, there were prominent gaps in the expression of neural progenitor markers, and excess neurons developed at the expense of OPCs. When Notch signaling is disrupted, excess neurons often differentiate at early stages at the expense of later-born cell types, including OPCs (Bingham et al., 2003; Haddon et al., 1998a; Itoh et al., 2003; Park et al., 2000; Shih and Keller, 1992). In the zebrafish embryo, Qiu et al. reported that Notch3 and Notch1a have redundant functions in controlling hindbrain patterning and neurogenesis (Qiu et al., 2009). They found that excess neurons developed in *notch1a* mutants that also had reduced *notch3* function (via morpholino injection), and rhombomere boundaries were not maintained. In combination, our analysis and the results of Qiu et al. suggest that *notch3* mutants have reduced OPCs in the early larval stage because of misallocation of the fates of some early neural progenitors (Qiu et al., 2009). Furthermore, the relatively mild and transient *notch3* mutant phenotype might reflect redundancy with *notch1a*. It is possible that, in the mouse, Notch3 has a similar role in OPC development that is masked by overlapping functions of other notch genes.

The elevated expression of *notch3*, *deltaD* and *gfap* and increased apoptosis in the CNS of *notch3* heterozygotes was a striking finding of our analysis. The elevation of progenitor markers suggests that the differentiation of some progenitors might be delayed by the partial reduction of Notch signaling in *notch3* heterozygotes. The elevated cell death in the heterozygotes suggests that some of these delayed progenitors undergo apoptosis, but the majority must differentiate properly because the pattern of neurons and OPCs is apparently normal and the fish are fully viable. The finding of changes in gene expression and cell death in the CNS specific to *notch3* heterozygotes suggests that a partial loss of Notch3 function could contribute to aspects of the pathophysiology of CADASIL that have not been observed in animals that completely lack *notch3* function.

Based largely on the temporal manifestation and detection of phenotypes, leukoaraiosis, subcortical ischemic events, mood disturbances, apathy and dementia have been thought to be secondary to vascular defects and reduced capillary density (reviewed in Hervé and Chabriat, 2010; Kalimo et al., 2002; Pantoni, 2010). Analysis of zebrafish *cloche* mutants, which lack endothelium but have normal hindbrain neuron clusters and axon tracts, provides evidence that endothelium is not required for proper patterning and organization of neurons in the early hindbrain (Ulrich et al., 2011). In zebrafish *notch3* mutants, OPCs are already reduced at stages when gross vascular patterning is intact, as assessed by the *Tg[fli1a:EGFP]* transgene, microangiography and endothelial markers. In addition, the OPC deficits are evident at stages when the other cell type of the vessel, the mural cell, has not been specified or undergone maturation (Miano et al., 2006; Santoro et al., 2009). Taken together, this indicates that *notch3* is likely to have parallel roles in regulating OPC development and *mbp* gene expression in larvae, and in promoting vascular integrity. Future investigation is required to identify the *notch3* targets involved in the abnormalities in OPC development and vasculopathy in brain and peripheral tissues, and to determine whether the brain phenotypes involve communication between neural and vascular cells, which has been proposed to account for the non-autonomous neuropathology in CADASIL phenotypes. Alternatively, in mouse and zebrafish, *notch3* is expressed widely in neural progenitors (Irvin et al., 2001; Lindsell et al., 1996; Qiu et al., 2009); therefore, the effects might instead be later manifestations of a *notch3* function in neural development rather than a secondary consequence of vascular defects.

Some zebrafish *notch3* mutants survive to adulthood. This is probably due to compensation by other notch family members such as *notch1a*, because adult mutants that are homozygous even for the presumed null *notch3^zm^* allele were recovered. Those *notch3* mutants that do survive show enlarged vessels in the telencephalon and accumulation of blood in peripheral vessels along the rostral caudal axis (in particular within the pectoral, anal and caudal fins), indicating loss of vascular tone within peripheral vessels. Cardiovascular development in zebrafish, as in mammals, involves specification of endothelial cell fates during the earliest stages of vessel development. Arterial differentiation is regulated by Shh, vascular endothelial growth factor (Vegf) and Notch signaling in zebrafish and in other vertebrates (Lawson et al., 2001; Lawson et al., 2002; Lawson and Weinstein, 2002a; Torres-Vázquez et al., 2003; Weinstein and Lawson, 2002). Complete loss of Notch signaling, as occurs in mutants for *mindbomb* (*mib*), an E3 ubiquitin ligase involved in Notch activation, results in deficits of arterial-specific and ectopic vein marker expression within the dorsal aorta (Lawson et al., 2002). Arterial and venous fates are intact in *notch3* mutant embryos and in adults, indicating that Notch3 is not involved or that other notch genes compensate for its loss.

In addition to endothelial cell specification, cardiovascular development in mammals and zebrafish involves recruitment of vascular mural cells (vMCs) during a process known as vascular myogenesis (Miano et al., 2006; Owens, 1995; Owens, 1998; Owens et al., 2004; Owens and Wise, 1997; Santoro et al., 2009). vMCs, VSMCs and pericytes initially act as support cells, but subsequently become highly specialized cells that control vasodilation and vessel caliber. During larval development in zebrafish, immature VSMCs are detected adjacent to the dorsal aorta by ultrastructural criteria and marker expression (Miano et al., 2006; Santoro et al., 2009). However, differentiated VSMCs are only apparent after 1 month of development, and definitive VSMCs, defined by dense plaques (hallmarks of a mature actin cytoskeleton), myofilament bundles and a basement membrane, are only apparent at 3 months (Miano et al., 2006; Santoro et al., 2009). Histological and ultrastructural analyses revealed vasodilation of both peripheral arteries and veins of *notch3* mutants, indicating that *notch3*, which is expressed in the walls of both vessel types, has an essential role in limiting vasodilation. Vascular smooth muscle cell contraction and relaxation regulate vessel caliber and local blood pressure in response to demand for oxygenated blood. Differentiated VSMCs display contractile properties, whereas undifferentiated VSMCs are proliferative (Owens, 1995). Pooling of blood and vasodilation in *notch3* mutants are consistent with a role for *notch3* in promoting the mature, contractile VSMC state to regulate vascular tone. *notch3* seems to play an additional role in regulating arterial architecture, because vessels positioned where arteries are typically located had thinner vessel walls, a morphology resembling that of veins rather than the typical thick cell walls of arteries, despite normal arterial venous patterning in vessels of embryos and adults. The absence of obvious patterning defects in embryos is consistent with adult-onset vasculopathy due to defective VSMC maturation or function in *notch3* mutants. Mural cells and VSMCs both contribute to the normal vascular response to Vegf in newly forming vessels and during regeneration (Carmeliet and Collen, 1999; Carmeliet and Storkebaum, 2002). We were unable to detect definitive pericytes associated with vessels in bony regions of mutant fin rays (where arteries reside in wild type); thus, impaired pericyte development or recruitment might contribute to impaired vascular integrity. Similarly, VSMC destruction associated with reduced pericytes has been reported in individuals with CADASIL (Kalimo et al., 2002; Ruchoux et al., 2003). Thus, zebrafish *notch3* mutant phenotypes show aspects of CADASIL pathology, specifically compromised VSMC and vascular integrity despite the different nature of the mutations in *notch3.*

Two *Notch3* knockout mice have been reported. In contrast to the sub-viable zebrafish mutants, both mouse knockout models are viable and fertile (Arboleda-Velasquez et al., 2008; Domenga et al., 2004; Kitamoto et al., 2005; Krebs et al., 2003). One *Notch3* knockout shows deficits in differentiation and maturation of VSMCs of small systemic vessels (Domenga et al., 2004). Similar to zebrafish *notch3* mutants, the tail arteries of this *Notch3* knockout are enlarged with a thin media reminiscent of veins. Domenga et al. suggested that Notch3 might act through a non-canonical pathway in VSMCs, because expression of classical Notch targets such as *Hes* genes were normal in *Notch3* mutants (Domenga et al., 2004). Instead, they hypothesized that Notch3 acts as a luminal pressure gauge or sensor that modulates the myogenic contractile response via regulation of the actin cytoskeleton (Domenga et al., 2004). In the second mouse knockout, ultrastructural vessel defects were not reported, but transcriptional profiling of purified populations of cerebral VSMCs did show deficits in regulators of muscle contraction, including regulators of cell motility and architecture and modest reductions of some canonical Notch targets, such as *Hes1* and *Hey1* in isolated VSMCs (Arboleda-Velasquez et al., 2008). Notably, the resting cerebral blood flow (CBF) in both *Notch3* mice was normal, but middle cerebral artery occlusion led to an exacerbated stroke phenotype. Specifically, CBF defects and more frequent ischemic lesions without vasoconstriction were observed, thus supporting a role for Notch3 in regulating contractile tone in cerebral vessels (Arboleda-Velasquez et al., 2011; Arboleda-Velasquez et al., 2008; Ayata, 2010; Domenga et al., 2004; Krebs et al., 2003). Similarly, evidence of compromised vascular tone and accumulation of blood in vessels in zebrafish *notch3* mutants was prominent in response to mild stress, but difficult to detect otherwise. Thus, *notch3* seems to have a conserved function in vascular myogenesis and vessel maturation via a mechanism involving canonical Notch pathway targets. Notably, in both zebrafish and mammals, VSMC differentiation is a Notch3-dependent process that occurs during post-larval (zebrafish) and postnatal (mammals) development. In contrast to mouse knockouts, some of the zebrafish *notch3* mutants do not survive to adulthood, perhaps owing to incomplete recovery from early CNS defects.

In summary, we have identified mutations disrupting *notch3* that cause oligodendrocyte deficits in embryos and vascular pathology in brain and peripheral tissues of adults, revealing roles for Notch3 in glia and vascular cells. It is thus possible that early glial deficits also contribute to white matter defects in individuals with CADASIL. Although arterial and venous fates are properly specified in embryos and adults, the fin vessels of adults show morphologies that are typical of veins. Moreover, adult vessels are swollen and show evidence of compromised vascular tone, including vasodilation and hemorrhage. Zebrafish *notch3* mutants will be valuable for future screens to identify Notch signaling modifiers or risk factors affecting the phenotype, and for studies of the activity of CADASIL mutant alleles and the signaling and cellular mechanisms contributing to vascular and other Notch3-associated pathologies.

## MATERIALS AND METHODS

### Ethics statement

The animal work performed in this study was approved by the Institutional Review Boards of Albert Einstein College of Medicine and Stanford University School of Medicine.

### Fish strains, mapping and positional cloning

#### Fish strains

Zebrafish embryos were raised at 28.5°C and staged according to Kimmel et al. (Kimmel et al., 1995). Identification of the *st51* mutation has been described (Pogoda et al., 2006). The *notch3^zm^* allele was purchased from Znomics.

#### Genetic mapping

Wild-type and *notch3^st51^* mutant larvae were sorted based on their *mbp* expression at 5 dpf. The map position of *notch3^st51^* was refined by scoring SSLP markers from linkage group 3 (LG3) in *notch^st51^* mapping crosses as described (Talbot and Schier, 1999). These mapping studies localized the *notch3^st51^* mutation to a region defined by the markers Z3725 and Z20058, which also contained the *notch3* gene (Woods et al., 2005). We sequenced the *notch3* gene to find the lesion.

#### Genotyping

To genotype the *notch3* alleles, individual larvae and adults were anesthetized in Tricaine as described and genomic DNA was prepared from adult fin samples or whole embryos and larvae (Westerfield, 1995).

The *notch3^st51^* allele was scored using the following primers to amplify fragments from genomic DNA: forward: 5′-GAGTGCCAGTCGAATCCCTGTC-3′; reverse: 5′-ACACTTGCAGGTGAAACCATTGAGC-3′. The PCR product was digested with *Rsa*I, which cuts the wild-type product but not the mutant.

Two different assays were used to genotype the *notch3^zm^* allele. In early experiments, we scored *notch3^zm^* by performing two separate PCR assays on each genomic DNA sample. One set of primers (5′-ACTCCGATCCGAAGTGAAGTAG-3′ and 5′-CAGGCAATCACATTAGGATGAA-3′) amplified a 184 bp product from the wild-type allele but no product from the mutant allele. The second set (5′-CTGAAGCCTATAGAGTACGAGCC-3′ and 5′-GTCCGAGCTGCAGAAGGAAATACTG-3′) amplified a product of about 700 bp from the mutant allele but no product from the wild-type allele. In more recent experiments, the following three primers were combined in single PCR reaction to genotype the *notch3^zm^* mutation: Common F 5′-GTCCGAGCTGCAGAAGGAAATACTG-3′, ZM R 5′-CTGTGCCTTATTTGAACTAACC-3′ and WT R 5′-GTGGAAACTCGATCCTACTCC-3′. A 283 nt product was amplified from the mutant allele, whereas the wild-type product was 190 nt.

#### RT-PCR

To examine the *notch3* mRNA in *notch3^st51^* mutants, RNA was prepared from the body of individual 3-day-old embryos using the Qiagen Rneasy Micro Kit. cDNA was synthesized using the SuperScript II First-Strand System (Invitrogen) with random hexamer primers following the manufacturer’s protocol. The tail of each embryo was used for genotyping as described above. PCR for *notch3* was performed on cDNAs with primers flanking the lesion site: forward 5′-GCACTTCAGGTACCAACTGTGA-3′ (across exons 11 and 12) and reverse 5′-AGAGCAAGGTGTGAGCAGTTCT-3′ (from exon 15). The different PCR products from wild-type, heterozygote and mutant cDNAs were run on an agarose gel and then cloned and sequenced. As a control, *ef1a* was analyzed as described (Monk et al., 2009).

For examination of Notch target expression, total RNA was isolated from wild-type and mutant fins and larvae at the specified stages using TRIZOL according to standard protocols. cDNA was reverse transcribed using SuperScript II reverse transcriptase (Invitrogen) and oligodT primers according to the manufacturer’s instructions. To control for genomic DNA contamination, reverse transcriptase was omitted using the same RNA samples. The following primers were used for qRT-PCR: *hey1f* 5′-CGATTTCAGCTCGTCGGACA-3′, *hey1r* 5′-ATGCAGAACCCCATGCCAAG-3′, *her9* (Yang et al., 2009), *her9f* 5′-CCAGCGTTTGCTTCTGCTACAAC-3′, *her9r* 5′-GCTCATTGCTTTCTGCTCCG-3′, *eef1a1l1(ef1α)f* 5′-AGCCTGGTATGGTTGTGACCTTCG-3′, *eef1a1l1(ef1α)r* 5′-CCAAGTTGTTTTCCTTTCCTGCG-3′.

qRT-PCR was performed using an Eppendorf Realplex3 machine according to standard protocols and cycling conditions. For *zm* genotypes: *n*=2 biological replicates and 3 technical replicates; for *st51* genotypes: *n*=5 biological and 3 technical replicates. Percent of wild-type expression was calculated using the ΔΔCT method using *ef1a* as the standard and wild type as the reference. *T*-test was calculated using Excel (Microsoft).

#### *In situ* hybridization

Whole-mount *in situ* hybridization was performed as described previously (Thisse and Thisse, 1998), except that for analysis of vascular markers and fins BM purple AP (Roche) was used as the substrate for alkaline phosphatase. To generate the *notch3* probe, a region of the gene corresponding to bp 14 to 935 of the *notch3* cDNA (GenBank accession number AF152001) was cloned into the pCRII-TOPO vector; the plasmid was digested with *Eco*RV and transcribed with SP6 RNA polymerase. *mbp* (Lyons et al., 2005), *olig2* (Park et al., 2002), *sox10* (Dutton et al., 2001), *deltaD* (Haddon et al., 1998b), *flt4* (Lawson et al., 2002), *efnb2a* (Durbin et al., 1998), *deltaC* (Smithers et al., 2000), *dab2* (Song et al., 2004) and *notch1a* (Bierkamp and Campos-Ortega, 1993) probes were prepared as previously described. For *gfap*, an 899 bp fragment of the gene was amplified from 72 hpf larval cDNA using the following primers: *gfap1*: 5′-GAGATGATGGGGCTAAACGA-3′; *gfap2*: 5′-TCCAGCAGCTTCCTGTAGGT-3′. The fragment was inserted into the TopoII (Invitrogen) vector; antisense probe was made by cleaving with *Xba*I and transcribing with SP6 RNA polymerase. For fin vessel analysis using *notch3* and *deltaC* probes, linear templates were amplified from cDNA using the following primers: *T7-notch3F* 5′-TAATACGACTCACTATAGGGGGTATTTCGAGACGCACGGC-3′, *T3-notch3R* 5′-CATTAACCCTCACTAAAGGGAATGTGTTGACACCATCGACGC-3′, *T3-deltaCF* 5′-CATTAACCCTCACTAAAGGGAAGGAGCACCTCAAACACCAGT-3′, *T7-deltaCR* 5′-TAATACGACTCACTATAGGGCACAACAGCATCCATCATCC-3′.

### Immunohistochemistry and cell death assays

Acetylated tubulin [1:1000 mouse α-acetylated alpha tubulin (Sigma, T6793) and 1:100 FITC-conjugated donkey α-mouse antibody (Jackson, 715-095-151)] and HuC/D (Molecular Probes) immunostainings were carried out as described previously (Lyons et al., 2008). Images were acquired on a Zeiss LSM confocal, Zeiss Observer equipped with Apotome, Perkin-Elmer spinning disk confocal or Leica SP2 confocal microscope. After imaging, larvae were genotyped for the *notch3* mutation.

Apoptosis was assessed via AO staining as described previously (Lyons et al., 2005).

*Tg[fli-EGFP]* transgenic larvae at the specified stages were visualized and imaged live or were fixed in 4% PFA and GFP signals were detected using an anti-GFP antibody. Briefly, 51–52 hpf embryos were fixed overnight (O/N) in 4% PFA and then dehydrated in MeOH via 25%/50%/75% MeOH in PBS. Larvae were rehydrated, treated with 10 ng/μl Proteinase K in PBSTri (PBS, 0.5% for Triton X-100) for 30 minutes and postfixed for 20 minutes in 4% PFA. Following washes with PBSTri, the embryos were preincubated for 3 hours in blocking buffer [10% fetal bovine serum (FBS) and 1% DMSO in PBSTri] before incubation with a 1:1000 dilution of chicken anti-GFP antibody (Invitrogen, A10262) in blocking buffer O/N at 4°C. The next day the embryos were washed with PBSTri and preincubated for 3 hours in blocking buffer. Following incubation O/N at 4°C with a 1:300 dilution of Alexa-Fluor-488-conjugated goat anti-chicken antibody (Jackson, 103-545-155) in blocking buffer, immunostained embryos were washed and then mounted in 1% low melting point agarose (Sigma, A9414) in a glass-bottom dish. Dorsal view images were obtained with a Leica SP2 confocal. After imaging, larvae were genotyped for the *notch3* mutation.

### Microangiography

Anesthetized 2-dpf progeny from clutches of *notch3^zm/+^* intercrosses were embedded in 1% low melting point agarose and 3–4 nl of 1% Fluorescein dextran (Invitrogen, D-1822) was injected into the sinus venosus, essentially as described previously (Weinstein et al., 1995). After injection, the larvae were cut out of the agarose and remounted under anesthesia (Tricaine) in 1% low melting point agarose on a glass bottom dish. The labeled vasculature was imaged in 60–63 hpf larvae using a Leica SP2 confocal microscope. After imaging, larvae were genotyped for the *notch3* mutation.

### Histology and transmission electron microscopy

Adults were anesthetized and the brains were dissected and fixed in 4% paraformaldehyde and embedded in paraffin. 10-μm sagittal sections were cut on a microtome, deparaffinized, and stained with H&E.

Fins from wild-type and *notch3* mutants were clipped according to standard protocols and fixed with 2.5% glutaraldehyde, 2% paraformaldehyde in 0.1 M sodium cacodylate buffer, postfixed with 1% osmium tetroxide followed by 2% uranyl acetate, dehydrated through a graded series of ethanol and embedded in LX112 resin (LADD Research Industries, Burlington, VT). Ultrathin sections (80 nM) were cut on a Reichert Ultracut UCT, stained with uranyl acetate followed by lead citrate and viewed on a JEOL 1200EX transmission electron microscope at 80 kv.

Toluidine-blue-stained sections (1 μM) were coated with permount solution, coverslipped, and images were acquired with an Axioskop2 microscope and Axiocam CCD camera (Zeiss). Images were processed in ImageJ, Adobe Photoshop and Adobe Illustrator.

### Quantification of vessel defects

The circumference of vessels within the bony regions of the fin rays (i.e. where arteries are positioned in wild type) was traced and the diameter was determined using ImageJ software. The distance between bony segments was measured using ImageJ (as depicted in Fig. 5A). The ratio of the artery/vessel diameter to length between the bone segments was calculated and plotted using Excel (Microsoft). ANOVA analysis was performed using the calculator at http://www.physics.csbsju.edu/stats/anova_pnp_NGROUP_form.html.

## Supplementary Material

Supplementary Material
