# Supplementary material for: notch3 is essential for oligodendrocyte development and vascular integrity in zebrafish
Source: Dis Model Mech. 2013 May 29;6(5):1246–59. doi: 10.1242/dmm.012005 (PMC3759344; doi:10.1242/dmm.012005)
Supplement: Supplementary Material [file supp_6_5_1246__index.html]

notch3 is essential for oligodendrocyte development and vascular integrity in zebrafish — notch3 is essential for oligodendrocyte development and vascular integrity in zebrafish — Supplementary Material 

# *notch3* is essential for oligodendrocyte development and vascular integrity in zebrafish

## 

**Files in this Data Supplement:**

- **Supplementary Material PDF**
