# Supplementary material for: notch3 is essential for oligodendrocyte development and vascular integrity in zebrafish
Source: Dis Model Mech. 2013 May 29;6(5):1246–59. doi: 10.1242/dmm.012005 (PMC3759344; doi:10.1242/dmm.012005)
Supplement: Supplementary Material [file supp_012005_DMM012005.pdf]

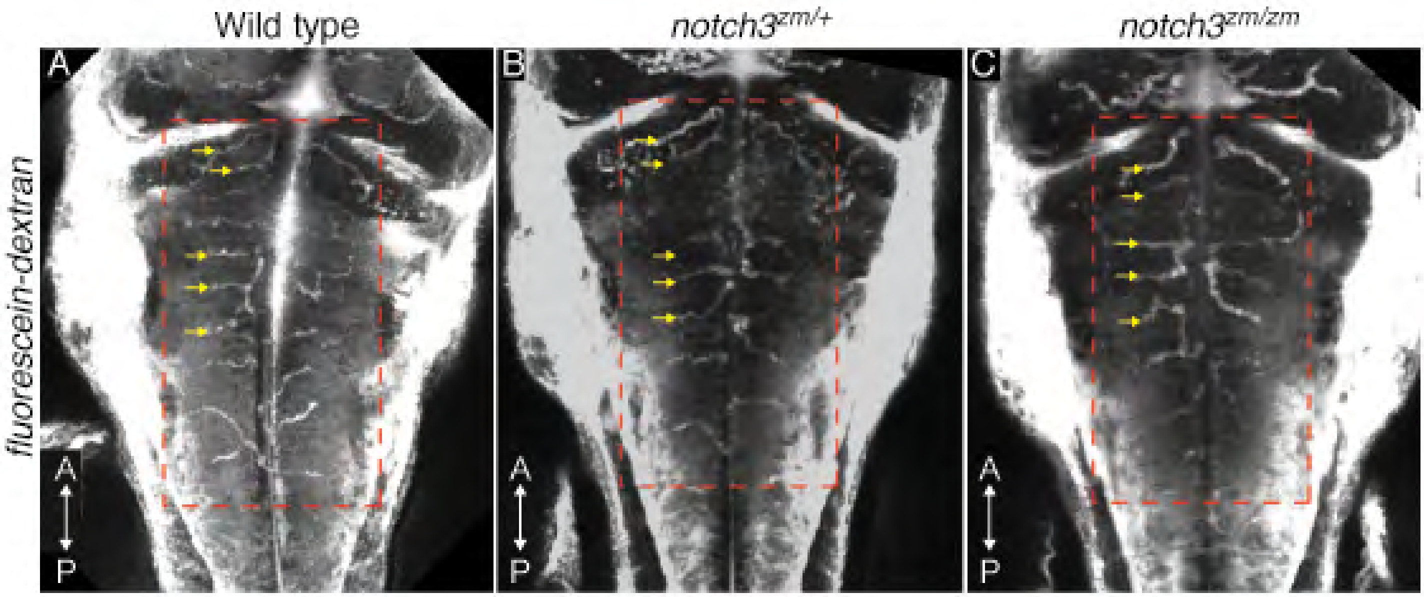

Figure S1. Microangiography of the vasculature in *notch3* mutants. Images of Fluorescein-dextran labeled vasculature of 60-63 hpf A) wild type (n=2), B) *notch3*$^{zm/+}$ (n=10) and C) *notch3*$^{zm/zm}$ (n=6) larvae reveals comparable patterning of the hindbrain vessels at the anatomical level (compare red dashed boxed areas). Yellow arrows indicate the Central arteries (CtAs). Images are max projections of dorsal views.

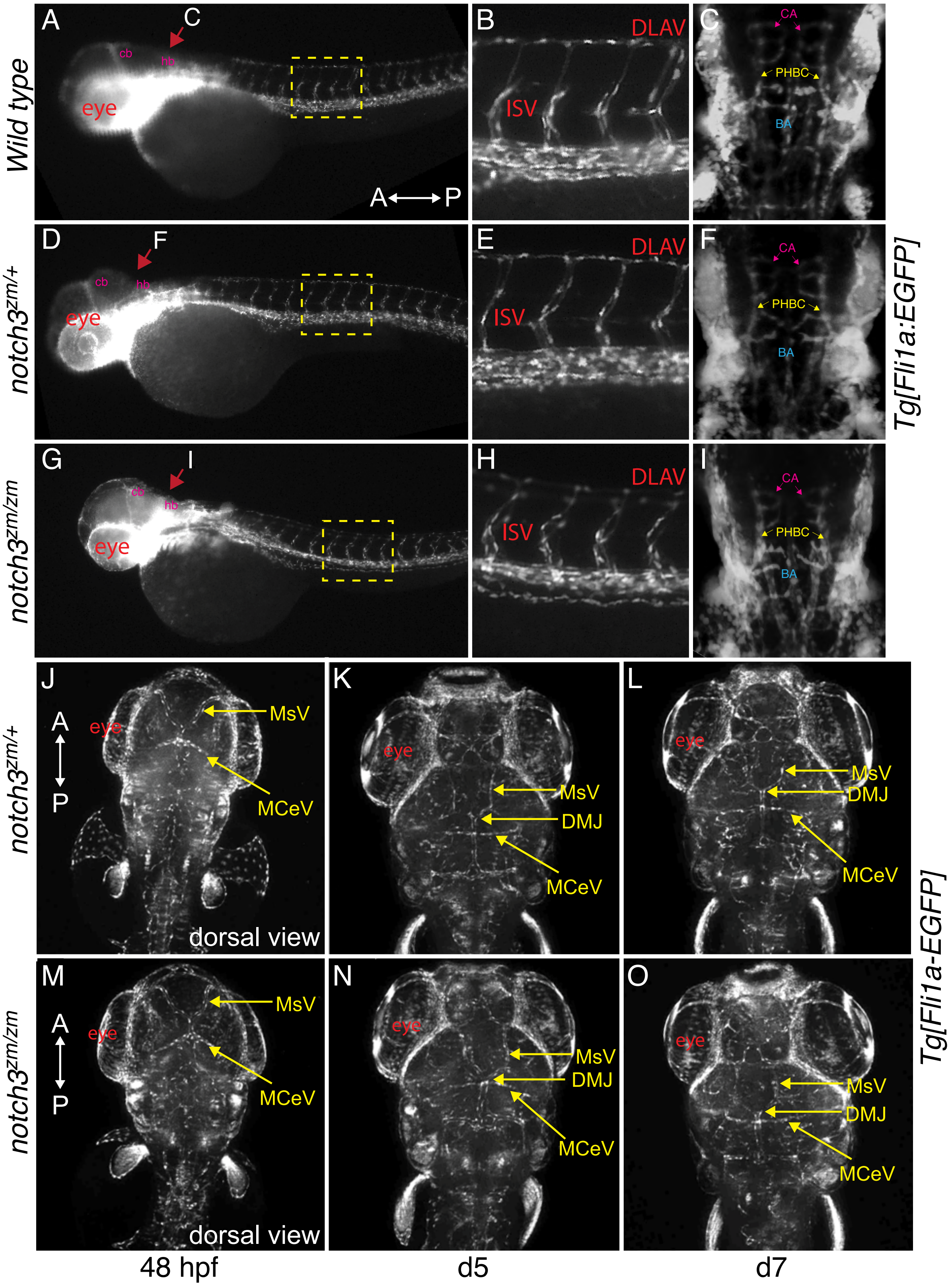

Figure S2. ***Tg[fli1a:EGFP]*** **labelled vasculature of A,B,C) wild-type, D,E,F)** ***notch3$^{zm/+}$*****, G,H,I)** ***notch3$^{zm/zm}$*** **were indistinguishable.** A-I) 52hpf. A,B,D,E,G,H) Lateral views; max projection images. Dorsal is to the top and the Anterior (A) and posterior (P) axis is as indicated. A,D,G) The yellow dashed boxes correspond to the regions shown in the higher magnifications in B,E,H. C,F,I and J-O) Dorsal views; max projection images of *Tg[fli1a:EGFP]* labelled vasculature system of C) wild type F, J-L) *notch3$^{zm/+}$* heterozygotes and I, M-O) *notch3$^{zm/zm}$* mutants. Vessel pattern at the anatomical level is intact in *notch3$^{zm/zm}$* mutants (48hpf=9 hets and 6 mutants; day 5=2 hets, 3 mutants; day 7 = 3 WT, 6 hets, 2 mutants). The orientation of the Anterior (A) and posterior (P) axis is indicated. DLAV indicates the Dorsal Longitudinal Anastomotic Vessel; ISV denotes the InterSegmental Vessels; CA indicates the central artery; PHBC denotes the primordial hindbrain channel; BA indicates the Basilar artery; MsV indicates the Mesencephalic Vein; DMJ denotes the Dorsal Midline Junction; MCeV indicates the Mid-Cerebral Vein.

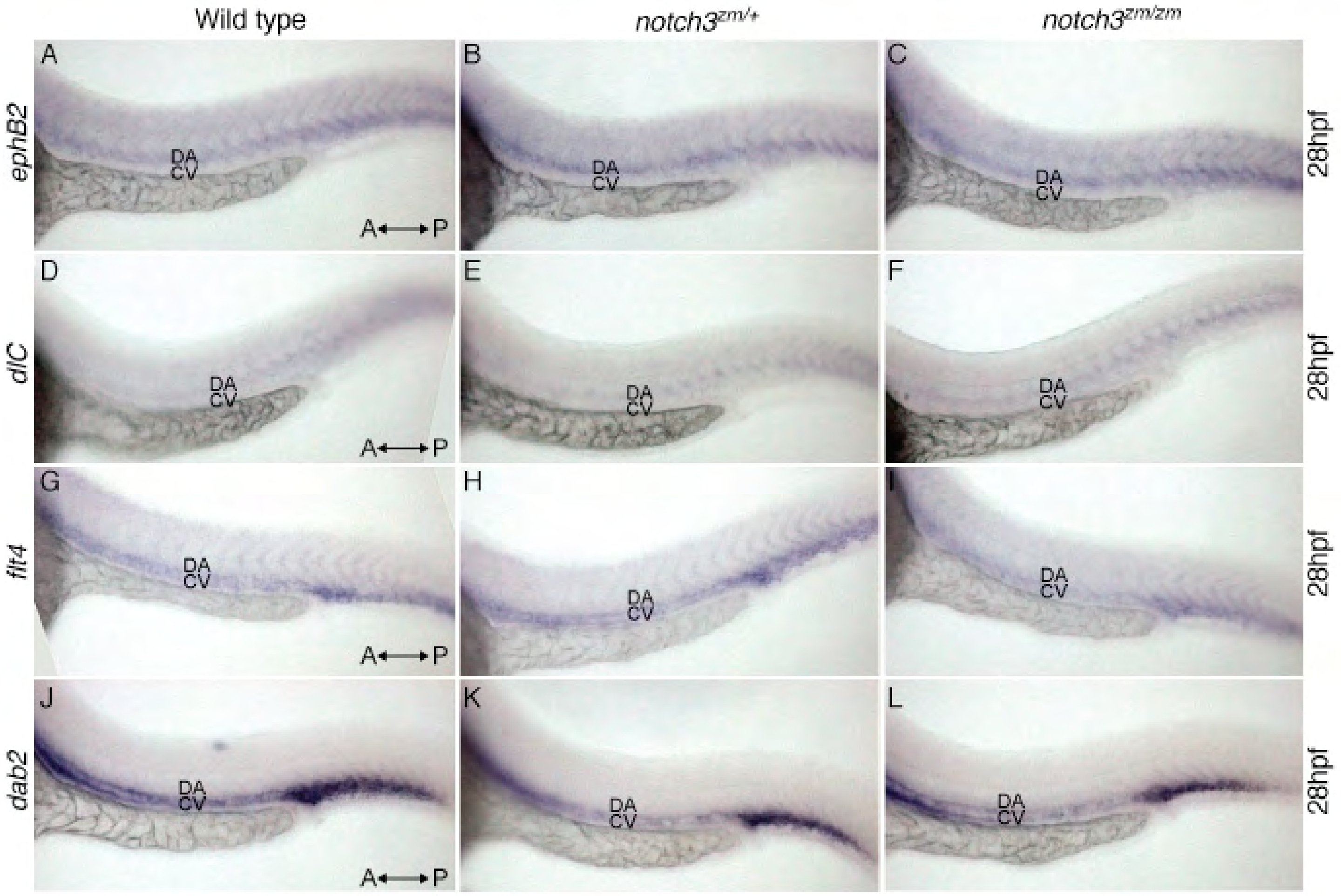

Figure S3. Expression of arterial and venous markers in *notch3* mutant larvae. Patterns of the arterial marker, A-C) *(efnb2a:* 3 wt, 9 hets, 4 mutants), D-F) *deltaC (dlc:* 1 wt, 8 hets, 6 mutants) and venous, G-I) *fms-related tyrosine kinase 4 (flt4:* 2 wt, 10 hets, 2 mutants) and J-L) *disabled homolog 2 (dab2:* 2 wt, 9 hets, 3 mutants) expression were indistinguishable between A,D,G,J) wild-type, B,E,H,K) *notch3*$^{zm/+}$ and C,F,I,L) *notch3*$^{zm/zm}$ mutants at 28 hpf. A-L) Dorsal is to the top and the orientation of the Anterior (A) and posterior (P) axis is as indicated. DA denotes dorsal aorta, CV indicates the cardinal vein.

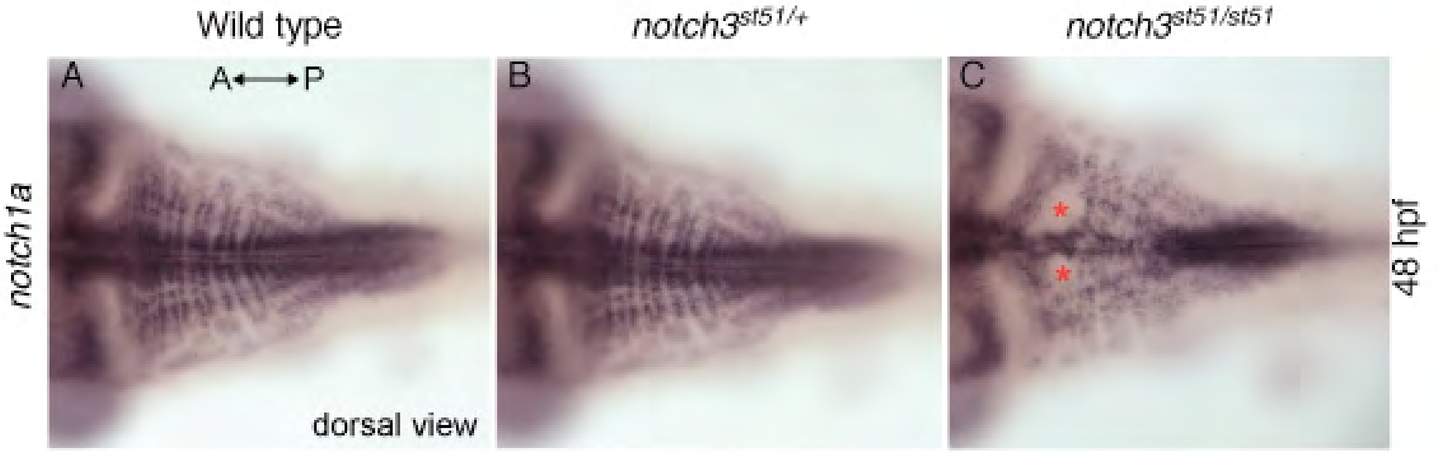

Figure S4. Expression of *notch1a* in *notch3* mutants. A) In wild-type and B) *notch3*$^{st51/+}$heterozygotes, and C) *notch3*$^{st51/st51}$ mutants *notch1a* transcripts were detected in the CNS, albeit with gaps in medial regions of *notch3*$^{st51/st51}$ mutants (asterisks). All panels are dorsal views, anterior (A) is to the left and posterior (P) is to the right.

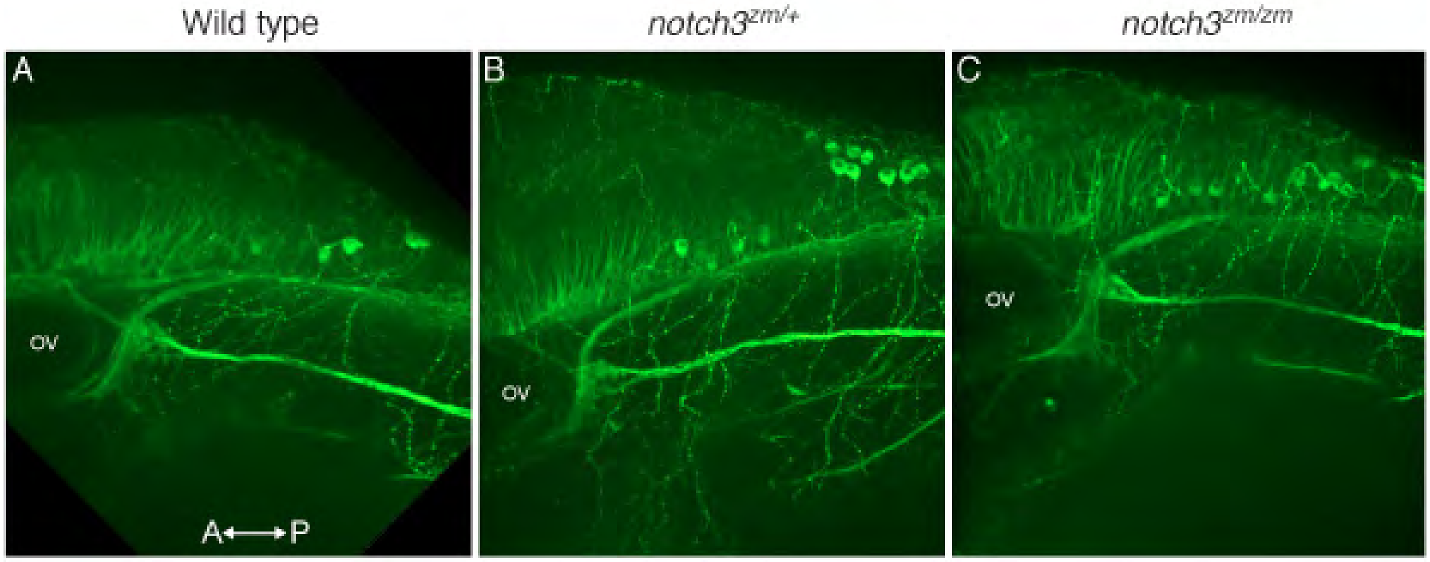

Figure S5. Axonal projections are intact in *notch3* mutants. Acetylated tubulin labeled axon tracts were comparable between A) Wild-type (n=4), B) *notch3*$^{zm/+}$heterozygotes (n=12), and C) *notch3*$^{zm/zm}$ mutant (n=7) larvae. Max projections of confocal images. Axes are as indicated. Ov denotes the otic vesicle.
